# Inferior ST-Elevation Myocardial Infarction Associated with Takotsubo Cardiomyopathy

**DOI:** 10.1155/2010/467867

**Published:** 2010-08-05

**Authors:** Oliver Koeth, Uwe Zeymer, Rudolf Schiele, Ralf Zahn

**Affiliations:** Department of Cardiology, Klinikum Ludwigshafen, Bremserstraße 79, 67063 Ludwigshafen, Germany

## Abstract

Takotsubo cardiomyopathy (TCM) is usually characterized by transient left ventricular apical ballooning. Due to the clinical symptoms which include chest pain, electrocardiographic changes, and elevated myocardial markers, Takotsubo cardiomyopathy is frequently mimicking ST-elevation myocardial infarction in the absence of a significant coronary artery disease. Otherwise an acute occlusion of the left anterior descending coronary artery can produce a typical Takotsubo contraction pattern. ST-elevation myocardial infarction (STEMI) is frequently associated with emotional stress, but to date no cases of STEMI triggering TCM have been reported. We describe a case of a female patient with inferior ST-elevation myocardial infarction complicated by TCM.

## 1. Introduction

Takotsubo cardiomyopathy (TCM) was primarily described in Japan and is usually characterized by transient left ventricular apical ballooning. TCM is named after the original Japanese octopus trap and shows a left ventricular dysfunction most commonly with preserved basal function and moderate-to-severe dysfunction in the midventricle or apical regions [1–4]. In a minority of patients a different pattern with preserved apical contractile function and impaired basolateral contractility was observed [1, 2, 5]. TCM predominantly affects women and is frequently triggered by preceding emotional or physical stress [6, 7]. The pathogenesis of the TCM is still unknown. Catecholamine-mediated cardiotoxicity provoked by emotional or physical stress has been proposed as an explanation [7]. Due to the clinical symptoms which include chest pain, electrocardiographic changes, and elevated myocardial markers, this syndrome may be misdiagnosed as an acute coronary syndrome [1, 2, 8]. Alternatively an acute occlusion of a large “wrap-around” left anterior descending coronary artery (LAD), extending to the inferior wall, can produce a typical Takotsubo contraction pattern [9]. ST-elevation myocardial infarction (STEMI) is frequently associated with emotional stress, but to date no cases of STEMI triggering TCM have been reported. We describe a case of a female patient with ST-elevation myocardial infarction complicated by TCM.

## 2. Case Presentation

**Figure 1:**
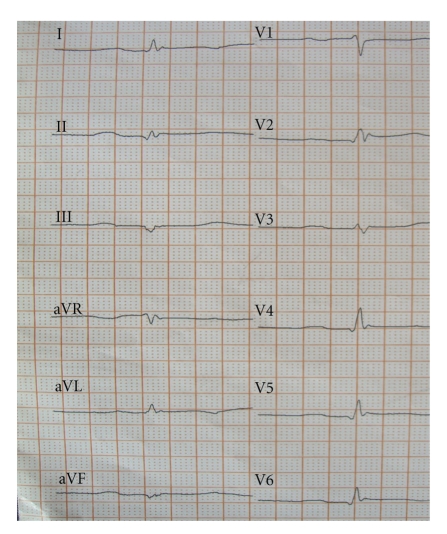
The initial electrocardiogram showed sinus rhythm and ST-elevations in the leads II, aVF, and V2–V6.

**Figure 2:**
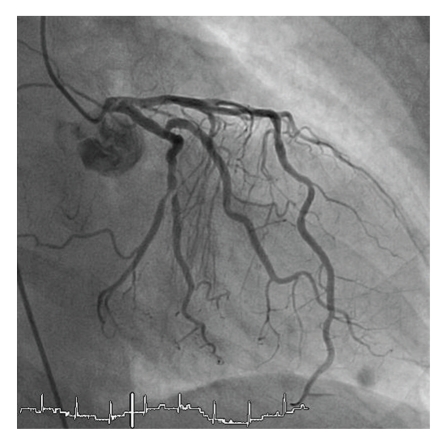
Coronary angiography revealing a 50% stenosis in the left anterior descending coronary artery and a subtotal occlusion (99%) of the circumflex coronary artery.

**Figure 3:**
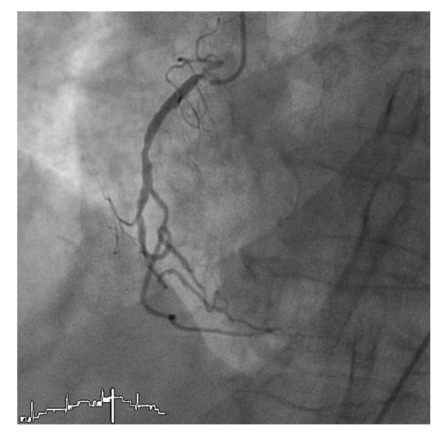
Coronary angiography revealing a 50% stenosis in the right coronary artery.

**Figure 4:**
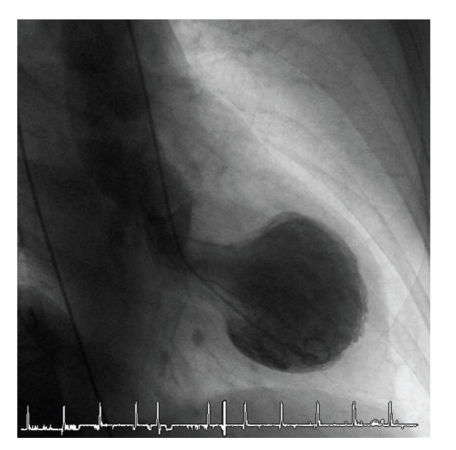
Angiogram showing a left ventricular dysfunction with preserved basal function and moderate-to-severe dysfunction in the midventricle and apical regions.

**Figure 5:**
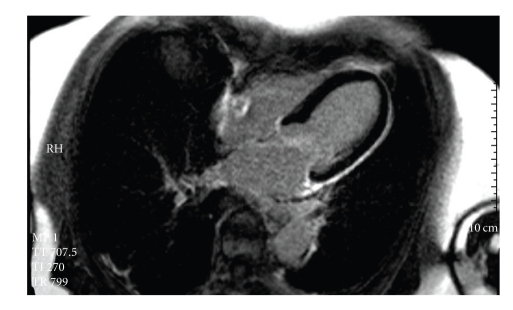
Cardiac magnetic resonance imaging showed a hyper-enhancement confined to the lateral wall.

An 82-year old female patient with a history of hypercholesterolemia and osteoporosis was admitted to the emergency department of a community hospital with chest pain. On admission she did not report about an obvious emotional stress situation. She was under chronic therapy with acetylsalicylic acid (100 mg/od), simvastatin (20 mg/od) and esomeprazole (20 mg/od). Initially she had a pulse rate of 68 beats/min and a blood pressure of 116/81 mmHg. Her physical examination was normal. The initial electrocardiogram showed sinus rhythm and ST-elevations in the leads II, aVF, and V2–V6 (Figure 1). ST-elevation myocardial infarction was diagnosed, and the patient received acetylsalicylic acid (500 mg i.v), clopidogrel (600 mg p.o.), and unfractionated heparin (5000 IE i.v.) and was immediately transferred for primary percutaneous coronary intervention (primary PCI) to our catheter laboratory. Time from symptom onset to primary PCI was 8 hours. The immediately performed coronary angiography revealed coronary three-vessel disease with a 50% stenosis in the LAD, a 50% stenosis in the right coronary artery, and a subtotal occlusion (99%) of the circumflex coronary artery (Figures 2 and 3). The left anterior descending coronary artery was not a large wrap-around vessel and did not supply the midinferior segment. We performed a primary PCI with bare metal stenting of the circumflex coronary artery (Coroflex Blue 2,5/8 mm). Angiogram showed a left ventricular dysfunction with preserved basal function and moderate-to-severe dysfunction in the midventricle and apical regions (Figure 4). Laboratory testing revealed elevated levels of Troponin T (0.56 ng/mL, (UNL:<0.03 ng/mL)) and creatinine kinase (214 U/L, (UNL:<145 U/L)). Catecholamine plasma levels were not measured. During the hospital stay the patient received fondaparinux (2,5 mg/od), beta blockers (metoprolol 47,5 mg/td), ACE inhibitors (ramipril 2,5 mg/td), acetylsalicylic acid (100 mg/od), clopidogrel (75 mg/od), statins (simvastatin 40 mg/od), and proton-pump inhibitors (esomeprazol 20 mg/od). Echocardiography on day 3, as well as contrast enhanced cardiac magnetic resonance imaging (CMI) on day 5, still showed a moderate reduced ejection fraction with typical apical ballooning. CMI showed a hyperenhancement confined to the lateral wall (Figure 5). A left ventricular hypertrophy, a dynamic LVOT obstruction, or a valvular heart disease was not observed on echocardiography or on CMI. ECG on day 7 showed a sinus rhythm and T-inversions in the leads V2–V6. Echocardiography on day 11 showed a mildly reduced ejection fraction. Wall motion abnormalities in the apical regions were not present anymore. Only an impaired lateral contractility could still be observed. Her recovery was uneventful, and she was doing well at discharge. She was discharged with a chronic medication including betablockers (metoprolol 47,5 mg/td), ACE-inhibitors (ramipril 2,5 mg/td), acetylsalicylic acid (100 mg/od), clopidogrel (75 mg/od), statins (simvastatin 40 mg/od), and proton-pump inhibitors (esomeprazol 20 mg/od).

## 3. Discussion

The pathogenesis of the TCM is still obscure. Catecholamine-mediated cardiotoxicity provoked by emotional or physical stress, multivessel coronary vasospasm and abnormalities in coronary microvascular function have been proposed as explanations [1, 2, 7]. Higher admission levels of plasma catecholamine in patients with TCM compared to patients with Killip class III myocardial infarction described by Wittstein and colleagues support that exaggeration of sympathetic stimulation is central to the cause of this syndrome [2, 7]. In addition, Takotsubo-like LV dysfunction was seen in patients with pheochromocytoma [10, 11]. In the present case the patient did not report about emotional stress preceding chest pain. Unfortunately plasma levels of catecholamines were not measured in this case. However, STEMI is frequently associated with emotional stress. TCM affects, like in the present case report, predominantly postmenopausal women [2]. The estrogen deficiency seems to play a major role in this syndrome. Due to the clinical symptoms which include chest pain, electrocardiographic changes, and elevated myocardial markers, TCM is frequently mimicking ST-elevation myocardial infarction in the absence of a significant coronary artery disease. Otherwise it has been reported that an acute occlusion of a large “wrap-around” LAD, extending to the inferior wall, can produce a typical Takotsubo contraction pattern [9]. Chao and colleagues [12] reported that the Takotsubo contraction pattern was found in a significant percentage of patients with acute LAD occlusion and an acute ST-elevation myocardial infarction. In this setting it has to be considered that angiography detects only atherosclerotic plaques that encroach on the lumen and may not detect lipid-rich plaques that have undergone positive remodeling [9]. Such plaques may not be detected on angiogram but carry the potential for transient clot formation and spasm. Therefore, LV stunning due to transient occlusion of the LAD as a part of the usual atherosclerotic process has to be considered as a reason for Takotsubo like LV dysfunction [12]. The subtotal occlusion of the circumflex artery explains only lateral and may be inferior wall motion abnormalities. However, the subtotal occlusion of the circumflex artery does not explain the severe dysfunction in the apical regions. In addition LV stunning would only be expected in the territory of the circumflex coronary artery (infarct vessel). Moreover the wall motion abnormalities in the apical regions disappeared after 11 days. Only an impaired lateral contractility (territory of the circumflex coronary artery/infarct vessel) could still be observed. In addition, only an isolated hyper-enhancement confined to the lateral wall (territory of the circumflex coronary artery/infarct vessel) was displayed on the MRI. Thus, transient clot formations in the LAD or in the right coronary artery were unlikely. Therefore, in the present case TCM was most likely triggered by an inferior ST-elevation myocardial infarction. To the best of our knowledge, this is the first case of STEMI associated with TCM.
